# Could the Use of an Enhanced Recovery Protocol in Laparoscopic Donor Nephrectomy be an Incentive for Live Kidney Donation?

**DOI:** 10.7759/cureus.889

**Published:** 2016-11-22

**Authors:** Aparna Rege, Harold Leraas, Deepak Vikraman, Kadiyala Ravindra, Todd Brennan, Tim Miller, Julie Thacker, Debra Sudan

**Affiliations:** 1 Surgery, Duke University Medical Center; 2 School of Medicine, Duke Univeristy Medical Center; 3 Division of Abdominal Transplantation, Duke University Medical Center; 4 Anesthesia, Duke University Medical Center

**Keywords:** enhanced recovery programs (erps), laparoscopic living donor nephrectomy, enhanced recovery after surgery (eras), length of stay (los), donor readmission rates, postoperative pain scores

## Abstract

Introduction and Background: Gastrointestinal (GI) recovery after major abdominal surgery can be delayed from an ongoing need for narcotic analgesia thereby prolonging hospitalization. Enhanced recovery after surgery (ERAS) is a multimodal perioperative care pathway designed to facilitate early recovery after major surgery by maintaining preoperative body composition and physiological organ function and modifying the stress response induced by surgical exposure. Enhanced recovery programs (ERPs) in colorectal surgery have decreased the duration of postoperative ileus and the hospital stay while showing equivalent morbidity, mortality, and readmission rates in comparison to the traditional standard of care. This study is a pilot trial to evaluate the benefits of ERAS protocols in living kidney donors undergoing laparoscopic nephrectomy.

Methods: This is a single-center, non-randomized, retrospective analysis comparing the outcomes of the first 40 live kidney donors subjected to laparoscopic nephrectomy under the ERAS protocol to 40 donors operated prior to ERAS with traditional standard of care. Our ERAS protocol includes reduced duration of fasting with preoperative carbohydrate loading, intraoperative fluid restriction to 3 ml/kg/hr, target urine output of 0.5 ml/kg/hr, use of subfascial Exparel injection (bupivacaine liposome suspension), and postoperative narcotic-free pain regimen with acetaminophen, ketorolac, or tramadol. Short-term patient outcomes were compared using Pearsons’s Chi-Squared test for categorical variables and the Kruskal-Wallis test for continuous variables. Additionally, a multivariate analysis was conducted to evaluate factors influencing patient length of stay and likelihood of readmission.

Results: ERAS protocol reduced the postoperative median length of stay decreased from 2.0 to 1.0 days (p=0.001). Overall pain scores were significantly lower in the ERAS group (peak pain score 6.0 vs. 8.00, p< 0.001; morning after surgery pain score 3.0 vs. 7.0, p=0.001; lowest pain score 0.0 vs. 2.0, p=0.016) despite the absence of postoperative narcotics. The average duration of surgery was shorter in the ERAS group (248 vs. 304 minutes, p<0.001). The average amount of intraoperative fluid used was significantly lower in the ERAS group (2500 ml vs. 3525 ml, p<0.001) without affecting the donor renal function. The incidence of delayed graft function was similar in the two groups (p=0.541). A trend toward lower readmission was noted with the ERAS protocol (12.8% vs. 27.5%, p=0.105). GI dysfunction was the most common reason for readmission.

Conclusion: Application of an ERAS protocol in a laparoscopic living donor nephrectomy was associated with reduced length of hospitalization and improved pain scores related likely to intraoperative use of subfascial Exparel and a shorter duration of ileus. Restricted use of intraoperative fluids prevents excessive third spacing and bowel edema, enhancing gut recovery without adversely impacting recipient graft function. This study suggests that ERAS has the potential to enhance the advantages of laparoscopic surgery for live kidney donation through optimizing donor outcomes and perioperative patient satisfaction.

## Introduction

Gastrointestinal (GI) recovery after major abdominal surgery can be delayed from an ongoing need for narcotic analgesia thereby prolonging hospitalization. Enhanced recovery after surgery (ERAS) is a multimodal perioperative care pathway designed to facilitate early recovery after major surgery by maintaining preoperative body composition and physiological organ function and modifying the stress response induced by surgical exposure [1]. Enhanced recovery programs (ERPs) in colorectal surgery have decreased the duration of postoperative ileus thus reducing the hospital stay while showing equivalent morbidity, mortality, and readmission rates in comparison to the traditional standard of care [2-3].

Laparoscopic living donor nephrectomy (LDN) has significantly transformed the outlook for individuals considering live kidney donation since first described by Ratner et al., in 1995 [4]. Despite benefits, the annual incidence of LDNs in the United States has decreased by nearly 15%, from 6573 in 2005 to 5630 in 2015, based on OPTN data as of November 11, 2016 [5]. Various reasons were explored for the decline in living donation [6]. However, transplant centers, federal health departments, and the community in general need to devise innovative solutions to remove barriers to living donation. One of the possible disincentives of LDN is the 16.8% incidence of complications [7] of which gastrointestinal (GI) dysfunction is the frequent indication for increased length of stay (LOS) and readmission rates. A 30% rate of Emergency Room visits/readmission was recorded at our center in 2014 largely from delayed GI recovery resulting from persistent narcotic use for postoperative pain management. Whether or not the ERAS pathway can further enhance the advantages of laparoscopic surgery and give incentive to donors by optimizing the perioperative satisfaction with regard to pain management, reduced length of stay and reduced rates of readmission still remains to be determined. Despite the potential benefits, the use of ERAS in LDN has been a rarity [8]. With this hypothesis in mind, we initiated a pilot trial to utilize a unique ERAS protocol in live kidney donors with the primary aim of reducing length of hospital stay and decreasing readmission rates.

## Materials and methods

This pilot trial is a single-center, nonrandomized, retrospective, quality-improvement project to evaluate the effectiveness of a full ERAS pathway in a laparoscopic, living-donor nephrectomy when compared with a historical cohort of patients undergoing laparoscopic donor nephrectomy with traditional standard of care. The ERAS pathway for LDN was implemented at our institute in September 2015 as part of a quality-improvement project, and from that time until present, all live kidney donors were subjected to the ERAS protocol during a laparoscopic nephrectomy. Subsequently, data was collected from 40 consecutive subjects undergoing LDN within the ERAS pathway from September 2015-October 2016 and compared with a prior cohort of 40 consecutive patients undergoing LDN with traditional standard of care from August 2014-August 2015.

### Living kidney donation standard of care pathway (SCP):

In this pathway, patients were kept nil per orally (NPO) for eight hours prior to surgery; no bowel preparation was used. Intraoperatively, sequential compressive devices (SCDs) were used for venous thromboembolic prophylaxis. Antibiotic prophylaxis was similar to the ERAS pathway. There were no set directions or protocols followed by anesthesia for intraoperative fluid management. Plans were individualized on a case-by-case basis. Intraoperative diuretics were used following fluid boluses with crystalloids or colloids if urine output was <0.5 ml/kg/hr. Perioperative pain management included intraoperative fentanyl boluses, postoperative IV ketorolac and IV fentanyl or Dilaudid, oral oxycodone, or Dilaudid as needed. Postoperative nausea vomiting (PONV) prophylaxis was the same as for the ERAS pathway.

### Living kidney donation Duke ERAS pathway:

Patients were educated about the pathway in the surgical clinic during a preoperative visit. Preoperatively, a bowel preparation was not routinely used. Patients were allowed clear fluids until two hours prior to the start of surgery and were given a carbohydrate drink, Clearfast®, two hours before surgery after which the NPO period of two hours begins. Table 1 provides details of the DUKE ERAS protocol for the various operative phases.

**Table 1:** Duke ERAS Pathway This table highlights the various details of the pre, intra, and postoperative aspects of the ERAS protocol implemented at Duke for laparoscopic living donor nephrectomy.

	PREOP HOLDING	INTRAOPERATIVE	POSTOPERATIVE
DIET	CHO drink 2 hrs. pre-op	NPO	Resume early diet
MULTIMODAL ANALGESIA	a. Acetaminophen 975 mg PO b. Gabapentin 600 mg PO	a. Fentanyl boluses b. Sub fascial Exparel (Bupivacaine liposome suspension) injection by surgeon c. Acetaminophen 1 g IV towards end of case d. Ketorolac 15 mg IV towards end of case	a. Acetaminophen PO b. Ketorolac IV (first 24 hrs.) c. Gabapentin PO d. PRN Tramadol PO
ANTIEMETICS	a. Scopolamine patch Emend for high risk PONV patients (failed scopolamine patch in the past)	a. Dexamethasone 4mg IV at start of case b. Zofran 4mg IV when closing	a. Scopolamine patch b. Zofran c. Phenergan (if needed)
VTE PROPHYLAXIS	Heparin 5000 Units SC	SCDs	SCDs Early ambulation
ANTIBIOTIC PROPHYLAXIS	Cefazolin 1-2 g IV or Clindamycin 600 mg IV (if allergic to Cefazolin)	repeat if procedure >4 hours	none

Intraoperatively, low-flow anesthesia was used with the gas flow at ≤1 L/min, and a 'goal-directed fluid therapy' with crystalloid infusion was infused at 3 ml/kg/hr. Fluid therapy was further guided by recording the stroke volume (SV) and stroke volume variation (SVV) using a non-invasive cardiac output monitor. Boluses of colloid were administered to optimize SV/SVV, with 250 ml of colloid bolus given over <15 min if SV increased by >10% and not required if SV increased by < 10%. The surgeon was alerted only when the urine output was < 0.5 ml/Kg/hour. No diuretics were used. A Foley catheter was removed at end of the case. For both pathways, every patient was scheduled for a one-week clinic follow-up visit at discharge.

Laparoscopic nephrectomy surgery was performed by the same three surgeons in the two cohorts. The surgical technique used was hand-assisted laparoscopy and was similar between the three surgeons. All three surgeons have been performing this surgery for over eight years, and there was no learning curve for this procedure in either cohort.

### Data collection

The study involves retrospective data collection from the electronic medical records for both groups including:

1. Patient demographics for both donor and recipient: age, gender, race, weight, body mass index (BMI), glomerular filtration rate (GFR) at donation, pre-operative baseline creatinine, and intraoperative narcotics. Donors were all healthy individuals without any pre-existing comorbidities.

2. Operative events: intraoperative fluids administered to the donor and intraoperative urine output measured for the donor and postoperative day (POD) 1 urine output measured for the recipient.

3. Total operative time calculated from time to incision to time to end of surgery.

4. Pain assessment: pain was assessed using a 0-10 verbal response scale (VRS), where “0” represents no pain, and “10” represents worst possible pain during every shift as part of the standard of care nursing protocol. The highest and the lowest pain score reported each day were recorded from the day of surgery until discharge. The set of maximum and lowest pain scores were averaged for each patient to give each patient an average maximum and average lowest pain score. Pain scores including the morning-after-surgery pain score (taken between 8 and 9 a.m.) and the peak and lowest pain score during the duration of hospitalization were collected.

5. Donor and recipient kidney function: based on donor baseline creatinine, postoperative day 1 and day 7 creatinine, recipient baseline creatinine and creatinine at discharge.

6. Incidence of delayed allograft function in the recipient.

7. Time to hospital discharge, i.e., length of stay (LOS).

8. Readmission rates including causes: all readmissions and emergency room (ER) visits for complications related to the LDN within 30 days of hospital discharge were recorded.

9. Any intraoperative events or complications.

Outcome Measures

The primary outcome for this study was the length of stay (LOS) for the donor. LOS is defined as the postoperative number of nights in the hospital. Secondary outcomes included operative times, operative fluid volume, postoperative pain scores, percent change in donor creatinine, donor readmission rates, recipient POD 1 urine output, incidence of delayed allograft function.

Statistical Analysis

Data was collected from the EPIC Maestro care and Innovian® databases for all patients undergoing LDN with traditional care and ERP pathway. Any missing data was obtained by chart review. Patient characteristics, including demographic, operative, and postoperative data elements were summarized with descriptive statistics and compared between the pre- and post-ERAS implementation groups. Demographics were compared between the two groups using Student's t-test. Analyses were performed using RStudio. (RStudio Team (2015), RStudio: integrated development for R RStudio, Inc., Boston, MA). Normally distributed continuous variables were compared with Student's t-test. For non-normal continuous variables, p-values were calculated by the Kruskal-Wallis test. Categorical variables were calculated using the Pearson chi-square test. For all statistical tests, p-values were two-tailed, and the alpha was set at 0.05.

The primary clinical outcome was LOS. The difference in LOS was assessed using the log-rank test. Secondary outcomes included readmission rates which were assessed with the Student's t-test. Multivariate regression analysis was performed for LOS and readmission rates. For comparisons of the other secondary efficacy endpoints such as operative times, operative fluids, percent change in donor creatinine, recipient day 1 urine output, incidence of delayed graft function, t-test, Chi-square test, and Fisher’s Exact test (for binary variables) as indicated were used. We also looked at the readmission rates of the outliers in each group for LOS.

Pain was assessed using a 0-10 verbal response scale (VRS), where “0” represented no pain and “10” represents worst possible pain and recorded during every shift as part of the standard of care nursing protocol. The morning-after-surgery pain score was recorded between 8 and 9 a.m. The highest and the lowest pain reported each day was recorded from the day of surgery until discharge. The set of maximum and minimum pain scores were averaged for each patient to give each patient an average maximum and average lowest pain score. Pain scores were compared between therapy groups using a rank-sum test.

## Results

### Demographics for both donors and recipients

A total of 79 live donor nephrectomies were performed during the study period (Aug 2014-Oct 2016), with 40 cases performed with the standard care protocol group (SCP) i.e., Group 1, and 39 cases in the ERAS group, i.e., Group 2. Both the groups were balanced since the baseline donor demographics were not significantly different between the two cohorts (Table 2), including age (SCP 45 vs. ERAS 47, p=0.416), weight (SCP166.8 vs. ERAS 173.2, p=0.337), body mass index (26.4 vs. 25.9, p=0.997), and GFR (SCP 104 vs ERAS 98, p=0.179).

**Table 2:** Baseline Characteristics of Donors * *Values are listed as percentage of patients meeting criteria with the total number of patients in parenthesis. Continuous variables are listed as median values with 95% CI in parenthesis.* Both groups appear balanced without any significant differences for all listed variables.

	Group 1(SCP) (N=40)	Group 2(ERAS) (N=39)	P-value
Age	45.0 (35.8-49.5)	47.0 (34.0-53.5)	0.416
BMI	26.4 (23.3-28.8)	25.9 (23.4-28.3)	0.977
Weight (lbs.)	166.8 (135.2-184.5)	173.2 (147.5-190.5)	0.337
Gender (Male)	67.5% (27)	69.2% (27)	0.869
Race			0.511
Asian	0.0% (0)	2.6% (1)	
Black	17.5% (7)	23.1% (9)	
Hispanic	7.5% (3)	2.6% (1)	
White	75.0% (30)	71.8% (28)	
GFR	104.0 (92.0-113.0)	98.0 (88.5-107.0)	0.179
Baseline Creatinine	0.8 (0.7-1.0)	0.8 (0.7-0.9)	0.7

Both groups were also balanced with regard to baseline characteristics of transplant recipients (Table 3), i.e., age, gender, race, dialysis status, ABO incompatibility, incidence of XM positivity, and diuretic use at transplant. Interestingly, recipient BMI was significantly higher in the ERAS group (27.0 vs. 30.6, p=0.011). Although this has no significance to this study, it highlights the fact that more obese recipients have undergone renal transplantation in the recent year.

**Table 3:** Baseline Characteristics of Recipients * *Values are listed as percentage of patients meeting criteria with total number of patients in parenthesis. Continuous variables are listed as median values with 95% CI in parenthesis.* RRT: renal replacement therapy. XM +ve: positive tissue crossmatch. Recipient BMI was significantly higher in the ERAS group (27.0 vs. 30.6, p=0.011)

*Variables*	Group 1 (SCP) (N=40)	Group 2 (ERAS) (N=39)	P-value
Age	50.5 (37.8-59.2)	55.0 (41.0-59.5)	0.726
BMI	27.0 (24.2-31.8)	30.6 (26.6-34.5)	0.011
Gender (Male)	40.0% (16)	33.3% (13)	0.539
Race			0.605
Black	17.5% (7)	17.9% (7)	
Hispanic	7.5% (3)	2.6% (1)	
White	75.0% (30)	79.5% (31)	
RRT	67.5% (27)	71.8% (28)	0.678
ABO Incompatibility	2.5% (1)	12.8% (5)	0.083
XM.+ve	5.0% (2)	5.1% (2)	0.979
Preoperative Creatinine	7.8 (5.2-10.2)	7.4 (5.6-10.9)	0.617
Warm Ischemia Time	29.0 (25.8-33.2)	29.0 (27.0-32.0)	0.796
Cold Ischemia Time	69.0 (51.5-103.5)	85.0 (64.5-138.0)	0.09
Lasix use	90.0% (36)	92.3% (36)	0.718
Mannitol use	100.0% (40)	87.2% (34)	0.019

### Outcomes

Our primary outcome was donor length of stay (LOS) after LDN. Group 1 (SCP) had a variable length of stay from 1-7 days, with an average stay of 2 days. For Group 2 (ERAS), the postoperative median length of stay reduced from 2.0 to 1.0 days (p<0.001). Table 4 highlights the donor outcomes.

**Table 4:** Outcomes for Donors * *Continuous variables are listed as median values with corresponding 95% CI in parenthesis. Values listed as percentage of patients meeting criteria have total number of patients in parenthesis.* For the donors within ERAS group LOS, operative times, operative fluid, and pain scores are significantly lower than SCP group, p<0.001.

Donor Variables	Group 1 (SCP) (N=40)	Group 2 (ERAS) (N=39)	P-value
Operative Times (minutes)	304.0 (266.0-336.8)	248.0 (228.0-269.0)	<0.001
Operative Fluid (ml)	3525 (3000-4425)	2500 (2000-2937.5)	<0.001
Operative Urine Output (ml)	793 (404-1130)	400 (290-500)	0.001
Baseline Creatinine	0.8 (0.7-1.0)	0.8 (0.7-0.9)	0.7
Creatinine Day 1 Post-op	1.5 (1.3-1.6)	1.5 (1.3-1.7)	0.876
Creatinine Day 7 Post-op	1.3 (1.1-1.5)	1.3 (1.1-1.5)	0.436
Percent Change in Creatinine Day 1 post-op	21.1 (0.0-42.9)	25.0 (5.6-35.4)	0.911
Percent Change in Creatinine Day 7 Post-op	50.0 (34.8-63.5)	38.8 (33.3-56.7)	0.149
Length of Stay	2 (1-7)	1 (1-1)	<0.001
Readmission	27.5% (11)	12.8% (5)	0.105
Morning After Donation Pain Score	7 (4-8)	3 (2-6)	<0.001
Peak Pain Score	8.0 (7.0-9.0)	6.0 (3.5-7.0)	<0.001
Low Pain Score	2 (0-3)	0 (0-2)	0.01

A univariate subset analysis was performed investigating if there was an association between the increased rate of readmission for donors with the increased LOS (Table 5). Donors were grouped on whether they had increased LOS beyond median or below the median. Although analysis does not suggest any such correlation, some other interesting points are brought forth. Lesser operative time (261 vs. 293 minutes) and lower operative fluids (2600 vs. 3375 ml) were significantly associated with shorter length of stay (1 vs. 2 days, p<0.001). Group 2 (ERAS) patients were more likely to have a shorter LOS (69.8%, p<0.001).

**Table 5:** Subset Analysis Based on LOS * *Values are listed as percentage of patients meeting criteria with total number of patients in parenthesis. Continuous variables are listed as median values with 95% CI in parenthesis.* Univariate analysis does not suggest any correlation between readmission rates and increased LOS, p=0.69. Lesser operative time (261 vs. 293 mins.) and lower operative fluids (2600 vs. 3375 ml) were significantly associated with shorter length of stay (1 vs. 2 days, p<0.001).

Variables	Above Median LOS (N=36)	Below Median LOS (N=43)	P-value
Group 2 (ERAS)	25.0% (9)	69.8% (30)	<0.001
Operative Time	293.0 (257.8 329.8)	261.0 (239.0-294.0)	0.024
Operative Fluid	3375 (3000-4053)	2600 (2000-3000)	<0.001
Operative Urine Output	793 (404-1130)	400 (290-500)	0.001
Baseline Creatinine	0.9 (0.7-1.0)	0.8 (0.7-1.0)	0.444
Creatinine POD 1	1.5 (1.3-1.6)	1.4 (1.2-1.7)	0.428
Creatinine at 1 week	1.3 (1.1-1.5)	1.3 (1.1-1.5)	0.932
Percent Change (Day 1)	22.2 (0.0-40.7)	28.6 (4.5-37.5)	0.922
Percent Change (Day 7)	47.2 (29.6-61.8)	41.4 (33.3-62.5)	0.769
Length of Stay (LOS)	2 (2-3)	1 (1-1)	<0.001
Readmission	22.2% (8)	18.6% (8)	0.69
Morning After Donation Pain Score	6.5 (4.0-8.0)	4.0 (2.0-6.0)	0.004
Peak Pain Score	8 (7-9)	6 (4-8)	<0.001
Low Pain Score	1 (0-2)	1 (0-2)	0.889

Median donor operative time was significantly shorter in Group 2 (ERAS) by 56 minutes compared to the standard protocol (248 (ERAS) v.s 304 (SCP) min, p<0.001) (Table 4). Median amount of intraoperative fluid used in the donor was significantly lower in the ERAS group (ERAS 2500 ml vs. SCP 3525ml, p<0.001), without affecting donor renal function in the form of percent change in donor serum creatinine (SCr) on postoperative day 1 (ERAS 25 vs SCP 21, p = 0.911) and on postoperative day 7 (ERAS 38.8 vs. SCP 38.8, p=0.149), and the recipient renal allograft function as documented by the recipient postoperative day 1 urine output (ERAS 6465.0 vs. SCP 5161.5, p=0.161). Overall pain scores were significantly lower in the ERAS group (peak pain score 6.0 vs. 8.0, p<0.001; morning-after-surgery pain score 3.0 vs. 7.0, p=0.005; lowest pain score 0.0 vs. 2.0, p=0.016) despite absence of narcotic use in the postoperative period in the ERAS group. 

A lower readmission rate was noted with the ERAS protocol (12.8% vs. 27.5%); although this was not statistically significant, p=0.105. In both groups, gastrointestinal complications (nausea, abdominal pain, constipation) were the most common reason for readmission. The incidence of delayed graft function was similar in the two groups (p=0.541). (Table 5). The kidney allograft function was not affected with reduced intraoperative fluids for the donor as shown by the similar incidence of urine on reperfusion in the two groups (SCP 100% vs. ERAS 92.3%, p=0.074) and comparable postoperative day 1 recipient urine output (SCP 5161 vs. ERAS 6465, p=0.161) (Table 6).

**Table 6:** Outcomes for Recipients * *Values are listed as percentage of patients meeting criteria with total number of patients in parenthesis. Continuous variables are listed as median values with 95% CI in parenthesis.* Recipients of allografts from ERAS donors had a similar incidence of urine on reperfusion (SCP 100% vs. ERAS 92.3%, p=0.074) and comparable postoperative day 1 recipient urine output (SCP 5161 vs. ERAS 6465, p=0.161). The incidence of delayed graft function was not different between the two groups, p=0.541.

Variables	Group 1 (SCP) (N=40)	Group 2 (ERAS) (N=39)	P-value
IV fluids	2925 (2400-3250)	2500 (1850-3000)	0.026
Urine on Reperfusion	100.0% (40)	92.3% (36)	0.074
Urine on Post-op Day 1	5161.5 (3378.8-7572.5)	6465.0 (4610.0-8035.0)	0.161
Discharge Creatinine	1.4 (1.1-1.7)	1.5 (1.2-2.0)	0.272
Creatinine Post-op 30 days	1.3 (1.1-1.6)	1.4 (1.2-1.5)	0.522
Percent Change in Creatinine (Discharge)	18.5 (14.8-27.5)	21.6 (14.4-29.0)	0.778
Percent Change in Creatinine (30 Days Post-op)	18.9 (12.7-25.7)	17.6 (12.9-21.3)	0.544
DGF	2.5% (1)	5.1% (2)	0.541

Finally, a logistic regression model was run to analyze donor readmissions (Table 7), but did not show any significant differences between the two groups; Group 2 OR 0.9 (0.71-1.14), p=0.39. Baseline creatinine appeared to be a protective variable, while creatinine on POD 1 appeared to be predictive of readmission for Group 1 (SCP); OR 1.85 (1.15-2.99), p=0.01.

**Table 7:** Logistic Regression for Donor Readmission Logistic Regression model run to analyze donor readmissions did not show any significant differences between the two groups, Group 2 OR 0.9 (0.71-1.14); p=0.39.

Variables	Odds Ratio	Lower 95% CI	Upper 95% CI	P-value
Group 2 (ERAS)	0.9	0.71	1.14	0.39
Age	1	0.99	1.01	0.61
BMI	1	0.97	1.02	0.81
Operative Time (minutes)	1	1	1	0.53
Operative Fluid (ml)	1	1	1	0.7
Baseline Creatinine	0.34	0.14	0.8	0.02
Creatinine on POD 1	1.85	1.15	2.99	0.01
Morning after surgery pain score	1.02	0.98	1.06	0.43
Length of Stay	0.96	0.87	1.06	0.38

## Discussion

Live kidney donation is one of the best solutions to satisfy the growing demand for kidneys for transplantation. However, fear of postoperative pain, prolonged hospitalization, and time off work are major disincentives for live kidney donation. Following the advent of laparoscopic donor nephrectomy (LDN), there was a 25% increase in individuals willing to donate kidneys to their loved ones [4]. Over the years, the annual incidence of LDN has decreased by nearly 15% from 6573 in 2005 to 5630 in 2015, possibly related to the incidence of gastrointestinal complications and length of stay [5]. Over the years, improvements in laparoscopic donor nephrectomy have revolved around optimizing pain control and accelerating recovery [9]. The widespread success of ERAS in major abdominal surgeries has created an opportunity to increase patient satisfaction in the live-kidney donor population by optimizing pain management and reducing length of stay. ERAS pathway has not yet been widely explored for laparoscopic living donation surgery. With successful implementation of the preoperative and intraoperative elements of an ERAS pathway for laparoscopic living donor nephrectomy, we have shown that early recovery, optimal pain control, and reduced hospital stay is within reach. Duke ERAS protocol significantly reduces the length of stay and minimizes the incidence of gastrointestinal complications with a somewhat lower tendency for readmissions or emergency room visits following LDN.

Of the various strategies adopted by ERAS to reduce the surgical length of stay and facilitate recovery, preoperative consumption of carbohydrate-containing clear liquids (predominantly in maltodextrin form) has provided significant benefits. Overnight fasting does not necessarily reduce gastric contents or raise the pH of gastric fluid, and, hence, the American Society of Anesthesiologists’ guidelines recommend intake of clear fluids until two hours prior to the induction of anesthesia [10]. Reducing the preoperative fasting period for clear fluids to two hours prior enhances patient comfort prior to surgery by reducing preoperative thirst, hunger, and anxiety without increasing the risk of pulmonary aspiration [11]. Carbohydrate-rich fluids have also been proven to reduce insulin resistance and patient catabolism, with a positive impact on perioperative glucose control and muscle preservation [12-13]. With the Duke ERAS protocol, we provide patients with Clearfast® which is a clear, preoperative drink containing complex carbohydrates including 12.5% maltodextrin, vitamins, minerals, electrolytes, and several amino acids that support an enhanced recovery. We also avoid routine mechanical bowel preparation which can contribute to preoperative dehydration in addition to creating significant patient discomfort. Thus, patients undergoing surgery within an ERAS protocol are less likely to be fluid responsive after the induction of anesthesia when compared with patients undergoing traditional fluid management preoperatively.

Intraoperative fluid balance potentially plays a crucial role in early postoperative mobilization and gastrointestinal recovery. The first goal of intraoperative fluid management should be ‘zero balance fluid therapy’ targeted at maintaining central euvolemia and avoiding excessive fluid administration which can result in ‘third spacing’ and tissue edema contributing to perioperative weight gain, bowel wall edema, and prolonged ileus [14]. Typical evaporative fluid losses during major abdominal surgery are 0.5-1 ml/kg/hr [15]. Maintenance fluid requirements should aim to maintain preoperative body weight, delivered with a 1-3 ml/kg/hr infusion of a balanced, crystalloid solution [16]. Several studies using individualized goal-directed fluid therapy (GDFT) utilizing a cardiac output monitor to optimize patients' stroke volume (SV) throughout the perioperative period have been shown to reduce complications after major surgery by 25-50% [17-20]. Positive fluid balance, on the contrary, has been shown to be associated with an increased incidence of acute kidney injury (AKI) after major surgery; whereas perioperative oliguria is not associated with renal dysfunction in laparoscopic surgery as the positive pressure pneumoperitoneum reduces renal blood flow thereby reducing fluid clearance [21].

The unique aspect of the Duke ERAS protocol is the inclusion of intraoperative GDFT using crystalloid infusion supplemented by colloid boluses to maximize donor SV/SVV. With this technique, there was a significant reduction in the amount of intraoperative fluids infused in the ERP group without any influence on the donor renal function in the form of intraoperative donor urine output, percent rise in donor creatinine on the 1st postoperative day, or any adverse impact on the allograft outcome. We also postulate that reduced intraoperative fluids may have indirectly helped in reducing operative times secondary to maintained tissue planes from reduced third-space fluid losses. All three of the surgeons performing the LDN have been doing this surgery for over eight years, and with the surgical technique remaining similar in the two cohorts, the possibility of the learning curve contributing toward the reduced operative times is negligible. Less tissue edema also could have potentially contributed to the early recovery of the postoperative bowel function, facilitating early discharge and reducing LOS.

The ERAS pathway is also built on multimodal pain-control strategies of which local anesthetics are a cornerstone. However, local anesthetics are relatively limited due to their shorter duration of action (≤ 7 hrs) [22]. Using DepoFoam, a multivesicular liposomal platform that encapsulates drugs without altering their molecular structure and then releasing them over a desired period of time, prolonged regional analgesia can be achieved [23]. Using this technology, Liposomal bupivacaine (Exparel; Pacira Pharmaceuticals, Inc., Parsippany, NJ) was recently approved by the U.S. Food and Drug Administration (FDA) for postsurgical analgesia to release bupivacaine slowly over 96 hours.  There has been enough literature evidence over the past ten years showing the efficacy of liposomal bupivacaine in statistically significant reduction of cumulative pain scores over 72 hours following wound infiltration after various surgical procedures [24]. The only other single-center ERAS study in the LDN population has used transversus abdominis plane block (TAP) with 0.5% Ropivacaine [7]. The Duke ERAS protocol utilizes Exparel, infiltrated intraoperatively by the surgeon at the subfascial level; with this we were successful in reducing overall pain scores in the ERP group as well as minimizing the overall increase in operating room time attributable to the addition of a TAP block as seen in the other series. Exparel also enabled a complete avoidance of postoperative narcotic pain regimens thus reducing the incidence of postoperative ileus. At discharge, patients did not receive any narcotic prescriptions further fortifying compliance with non-narcotic pain management.

There are several limitations to this study. Due to the small number of patients included in this study, this study was not powered to identify significant differences in the rate of readmissions between the two groups. Since this was a quality-improvement project in which we implemented a perioperative protocol that has been proven to reduce the length of stay and optimize postoperative recovery in other abdominal surgeries, we did not consider conducting a randomized control trial to test our hypothesis. However, we did consider comparing outcomes between two centers performing LDN with different care pathways, i.e., SCP vs. ERP, but did not pursue this considering several confounders including different standards of perioperative care, surgeon expertise, surgical techniques, and etc. could have impacted the results. Further, we do admit that knowledge of the donor being cared in the ERP could have possibly influenced early discharge in the ERP cohort. Pain scores as such could not be validated due to the variability in the patient interpretation of pain and inconsistent timing for recording of the scores. Implementing reliable pain scales and consistent times for recording the scores could possibly resolve any bias associated with the differences in pain scores between the two cohorts. Lastly, there is a lack of data in the literature to support or refute the use of liposomal bupivacaine administered as a peripheral nerve block for the management of postoperative pain. It would be interesting to further study outcomes of this study using a similar ERP without Exparel but with the use of routine local anesthetic agents injected subcutaneously to identify if the benefit of ERP is a result of GDFT or Exparel or a combination of both. We will be addressing most of these issues along with an additional survey of patients in the follow-up period to record patient reported outcomes and satisfaction in a subsequent larger prospective randomized control trial versus a multicenter trial.

## Conclusions

Application of a special ERAS protocol in laparoscopic living donor nephrectomy was associated with a reduced length of hospitalization. Improved pain scores resulted from a combination of the intraoperative use of subfascial Exparel and a reduced incidence of postoperative ileus with a complete avoidance of narcotic pain regimens. Restricted intraoperative fluids reduced the operative times by promoting clarity of surgical planes with prevention of excessive third spacing without adversely affecting allograft outcomes. GDFT helped reduce bowel edema, thereby enhancing postoperative gut recovery. Although readmission rates were not statistically significant, a larger study may further clarify this result. This study suggests that use of ERAS may enhance advantages of laparoscopic surgery for live kidney donation through optimizing donor outcomes and improved perioperative patient satisfaction. Regular application of ERPs in the future could incentivize donors by decreasing overall postoperative complications and facilitate earlier return to work.
